# Identification of a second gene associated with variation in vertebral number in domestic pigs

**DOI:** 10.1186/1471-2156-12-5

**Published:** 2011-01-14

**Authors:** Satoshi Mikawa, Shuji Sato, Masahiro Nii, Takeya Morozumi, Gou Yoshioka, Noriaki Imaeda, Tsuneko Yamaguchi, Takeshi Hayashi, Takashi Awata

**Affiliations:** 1 Animal Genome Research Unit, National Institute of Agrobiological Sciences, Tsukuba, Ibaraki 305-8602, Japan; 2 Livestock Research Institute, Tokushima Agriculture, Forestry and Fisheries Technology Support Center, Kamiita, Tokushima 771-1310, Japan; 3 STAFF Institute, Tsukuba, Ibaraki 305-0854, Japan; 4 Gifu Prefecture Livestock Research Institute, Minokamo, Gifu 505-0037, Japan; 5 Chiba Prefecture Livestock Research Center, Yachimata, Chiba 289-1113, Japan; 6 National Livestock Breeding Center, Odakura, Nishigo, Fukushima 961-8511, Japan; 7 National Agricultural Research Center, Tsukuba, Ibaraki 305-8666, Japan

## Abstract

**Background:**

The number of vertebrae in pigs varies and is associated with body size. Wild boars have 19 vertebrae, but European commercial breeds for pork production have 20 to 23 vertebrae. We previously identified two quantitative trait loci (QTLs) for number of vertebrae on *Sus scrofa *chromosomes (SSC) 1 and 7, and reported that an orphan nuclear receptor, *NR6A1*, was located at the QTL on SSC1. At the *NR6A1 *locus, wild boars and Asian local breed pigs had the wild-type allele and European commercial-breed pigs had an allele associated with increased numbers of vertebrae (number-increase allele).

**Results:**

Here, we performed a map-based study to define the other QTL, on SSC7, for which we detected genetic diversity in European commercial breeds. Haplotype analysis with microsatellite markers revealed a 41-kb conserved region within all the number-increase alleles in the present study. We also developed single nucleotide polymorphisms (SNPs) in the 450-kb region around the QTL and used them for a linkage disequilibrium analysis and an association study in 199 independent animals. Three haplotype blocks were detected, and SNPs in the 41-kb region presented the highest associations with the number of vertebrae. This region encodes an uncharacterized hypothetical protein that is not a member of any other known gene family. Orthologs appear to exist not only in mammals but also birds and fish. This gene, which we have named *vertnin *(*VRTN*) is a candidate for the gene associated with variation in vertebral number. In pigs, the number-increase allele was expressed more abundantly than the wild-type allele in embryos. Among candidate polymorphisms, there is an insertion of a SINE element (PRE1) into the intron of the Q allele as well as the SNPs in the promoter region.

**Conclusions:**

Genetic diversity of *VRTN *is the suspected cause of the heterogeneity of the number of vertebrae in commercial-breed pigs, so the polymorphism information should be directly useful for assessing the genetic ability of individual animals. The number-increase allele of swine *VRTN *was suggested to add an additional thoracic segment to the animal. Functional analysis of *VRTN *may provide novel findings in the areas of developmental biology.

## Background

In mammals, the vertebral formula shows developmental constraint [1]. The number of cervical vertebrae is fixed at 7, and the total number of thoracic and lumbar vertebrae tends to be 19, although the specific counts vary among species. For example, in the Monotremata, Marsupialia, Lagomorpha, Rodentia, and Artiodactyla, the total number of thoracic and lumbar vertebrae is conserved at 19, which is thought to be the primitive form. In comparison, this number is increased in the Perissodactyla (e.g., horse, 24 vertebrae) and Carnivora (e.g., dog, 20 vertebrae) and is reduced to 17 in the Primata. However, these changes are lineage specific, and variation is restricted within each species, as is seen in the Primata [2].

Wild boars, which are the ancestors of modern domestic pigs, have 19 vertebrae. In comparison, European commercial breeds have increased numbers ranging from 20 to 23 [3]. These breeds have long been selectively bred for enlargement of body size in order to increase meat production and improve reproductive performance. This process has presumably increased the number of vertebrae.

In previous papers on studies of F_2 _families, we reported two quantitative trait loci (QTLs) for number of vertebrae; the loci were found on *Sus scrofa *chromosomes (SSCs) 1 and 7 [4-6]. These two QTLs acted independently, and each had a mainly additive effect (approximately 0.55 and 0.60 per allele, respectively). In the F_2 _families, wild boar and Asian local breeds had the wild-type alleles at both QTLs. All the alleles of European breeds at the QTL on SSC1 increased the number of vertebrae, but only some of the alleles of European breeds at the QTL on SSC7 increased vertebral number. At the QTL on SSC1, we found a 300-kb region fixed in a variety of European commercial breeds; the gene encoding an orphan nuclear receptor/germ cell nuclear factor (*NR6A1*/*GCNF*) was located in this region [7]. The European allele of *NR6A1*/*GCNF *had a nonsynonymous substitution (C→T at nucleotide 748 of AB248749; Pro192Leu) that led to three times the binding activity to cofactors (NCOR1 and RAP80) as with the wild-type allele product. However, genetic variation of *NR6A1*/*GCNF *was not detected in today's commercial-breed pigs, and they have no fewer than 20 vertebrae. Interestingly, the number-increasing-type (Leu-type) alleles were also detected in some Chinese indigenous breeds, in which introgression of Western germplasm has occurred with aim of improving their productivity and the number of vertebrae has increased [8].

In the current report, we describe our map-based study of the QTL on SSC7, which was responsible for the variation in vertebral number in the today's commercial-breed pigs and may affect many phenotypic traits. Near the QTL for vertebral number on SSC7, other QTL effects for body composition [5,6], growth rate [9,10], and fat deposition [11,12] were also detected. We expect that our identification of the gene at this QTL will be of great benefit to the pork production industry.

## Results

### Defining the QTL region in a Large White population

**Figure 1:**
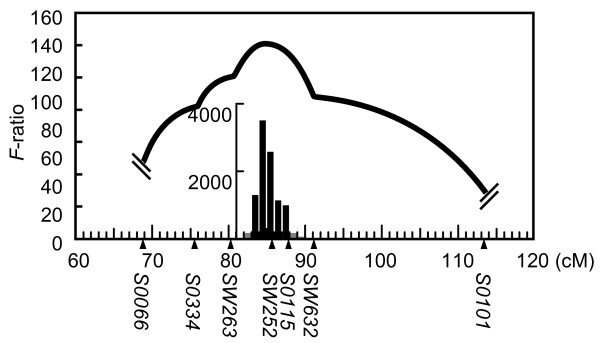
**Interval mapping of the QTL for number of vertebrae on SSC7**. A QTL analysis was performed in a JD family consisting of a Duroc sire, five Chinese Jinhua dams, six F_1 _sires, 21 F_1 _dams, and 528 F_2 _progeny. Plots of the *F*-ratio for interval mapping analysis are shown. The 95% confidence interval was analyzed with 10,000 repeats of bootstrap samples by QTL Express software and is indicated in the inset bar graphs. Black bars mean that the regions were included within 95% of samples. The 95% confidence interval for this QTL was from 83 cM to 88 cM on SSC7, between markers *SW263 *and *S0115*. In this analysis, a linkage map constructed by using the JD family was used.

We first developed 54 microsatellite markers in the 95% confidence interval (approximately 5 cM, or 9 Mb; Figure 1) of the QTL on SSC7 by using a comparative gene map for human and pig [13]. We also used swine genome draft sequences, Sscrofa9 published by the International Swine Genome Sequencing Consortium (SGSC; [14]) for the assignment of the markers to the swine genome (Additional file 1 Figure S1; Additional file 2 Table S1; Additional file 3 Table S2).

We next evaluated the QTL types of sires and dams in a closed breeding population of Large White (Awa-York, AY population) by using half-sib analysis of genotype and phenotype data for approximately 1100 progeny. We found nine heterozygotes that had the wild-type allele (wt) and the allele associated with increased numbers of vertebrae (number-increase allele, Q). We also found six homozygotes: five had the number-increase type (Q/Q) and one had the wild type (wt/wt), as judged by *Z*-score test [15] and multiple comparison test (Table 1). We therefore had 19 Q alleles and 11 wt alleles.

**Table 1:** Half-sib analysis of QTL on SSC7 in a Large White population (AY population)

	Average no. of vertebrae (no. of offspring)				**QTL type (haplotype of 57 markers **^**a**^**)**	
						
	Left homolog inherited	Right homolog inherited	***t*-test **^**b**^	***Z*-test **^**c**^	Left	Right
Sire						
1606	21.94 (83)	21.43 (84)	**		Q (d)	wt (f)
5901	22.07 (56)	21.54 (50)	**		Q (a)	wt (f)
503	21.86 (44)	21.49 (55)	**		Q (d)	wt
5910	21.95 (40)	21.44 (46)	**		Q	wt
5204	21.96 (46)	21.50 (48)	**		Q (a)	wt (e)
9305	21.82 (15)	21.57 (12)	**		Q (b)	wt
3605 ^d^	21.90 (31)	21.89 (37)		-6.9	Q	Q
3705	21.81 (64)	21.36 (67)	**		Q	wt
5002 ^d^	21.82 (60)	21.81 (64)		-7.9	Q (a)	Q
Dam						
1308	21.90 (10)	21.25 (8)	**		Q (c)	wt (e)
1402 ^d^	21.80 (25)	21.71 (21)		-2.6	Q (a)	Q
9404 ^d^	22.00 (22)	21.94 (23)		-3.5	Q (a)	Q (d)
2904	21.92 (13)	21.33 (12)	**		Q (b)	wt
8502 ^d^	21.83 (18)	21.80 (15)		-3.0	Q	Q (c)
2206 ^d^	21.58 (19)	21.57 (21)		-2.4	wt (e)	wt

The two homologous chromosomal regions for each individual are shown arbitrarily as Left or Right. Q and wt mean the vertebral-number-increase allele and the wild-type allele, respectively.

^a ^The same letters indicate identical haplotypes of the 57 markers within the 95% confidence interval.

^b ^Heterozygosity was judged by *t*-test (** means *P *< 0.01).

^c ^Homozygosity was judged by *Z*-score < -2.0, in which the Q-to-wt substitution effect was 0.49, as calculated with the data from nine heterozygotes in the Large White population.

^d ^QTL type (Q/Q or wt/wt) was judged by multiple comparison analysis of vertebral number.

**Figure 2:**
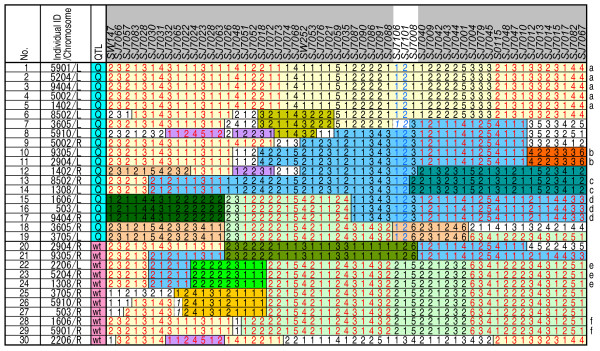
**Haplotype analysis in the 95% confidence interval of the QTL on SSC7 in AY population**. Haplotypes of microsatellite markers were analyzed in the nine sires and six dams in which allele types at the QTL (number-increase-type: Q or wild-type: wt) were determined. Microsatellite markers were genotyped with sires, dams, and their progeny, and haplotypes were reconstructed. The two homologous chromosomal regions for each individual are shown arbitrarily as Left (L) or Right (R). Numbers in each marker column represent allele types. Blocks of the same color are from the same IBD (identical-by-descent) region, which was supported by more than four markers. Italic characters indicate ambiguous alleles in determination of IBD regions. Blue letters indicate the haplotype of *SJ7106 *and *SJ7101 *conserved in the Q alleles. Red letters indicate distribution in the chromosomal regions of both the Q and the wt alleles. The candidate region contained three markers (*SJ7106*, *SJ7101*, and *SJ7008*) (white background in header row of table). Letters that are the same at the right of the panel indicate haplotypes that were identical at all 57 markers.

For these 30 alleles in 15 animals, the haplotypes of 57 microsatellite markers, including the 54 novel markers, were determined within the 95% confidence interval (Figure 2). We first searched haplotypes of some of the markers fixed within the 95% confidence interval of the Q allele, and found that a haplotype of two markers *SJ7106 *and *SJ7101 *was conserved (allele types 1 and 2 in Figure 2). We next searched for identical-by-descent (IBD) regions distributed in the 95% confidence interval of both the Q and the wt alleles. Because we considered these IBD regions to be inconsistent with the phenotypic variation, we excluded them from the candidate region. In this case, the candidate region was narrowed between *SJ7088 *and *SJ7040*, and contained the two markers *SJ7106 *and *SJ7101 *described above and *SJ7008 *(Figure 2).

**Figure 3:**
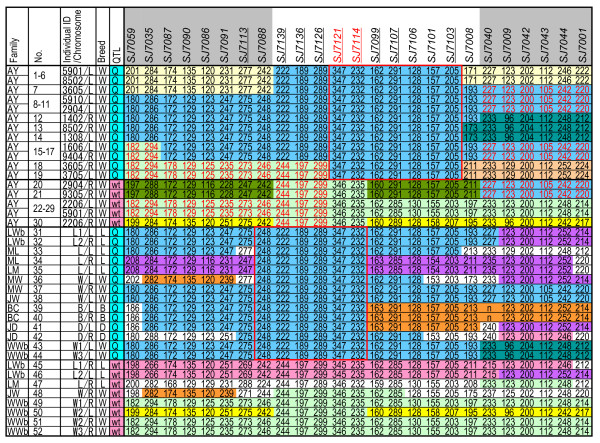
**Detailed haplotype analysis in AY population and parent pigs of F_2 _families**. Nine novel microsatellite markers (underlined) were developed from the sequences of BAC clones, CH242-92H3 and CH242-154M12. These markers were added to the haplotype analysis of the AY population. In this population, some of the genetic intervals between *SJ7059 *and *SJ7001 *had identical haplotypes, and two individuals are shown for each haplotype: 5901/L and 8502/L for allele types no. 1 to no. 6; 5910/L and 2904/L for allele types no. 8 to no. 11; 1606/L and 9404/R for allele types no. 15 to no. 17; and 2206/L and 5901/R for allele types no. 22 to no. 29. Numbering is the same as in Figure 3. Haplotype analysis was also performed in the parent pigs of the F_2 _families of our previous study, in which the QTL types of the parent pigs had been determined (Lower part). Numbers in each marker column are the sizes of alleles as amplified DNA fragments (in bp). Red boxes indicate conserved regions detected in the 95% confidence interval for the number-increase (Q) allele in the AY population and in the parent pigs of the F_2 _families. Markers common to the two conserved regions are indicated in red letters (i.e. *SJ7121 *and *SJ7114*). The QTL was likely to be located between the flanking markers (*SJ7126 *and *SJ7099*). Breeds are: W, Large White; L, Landrace; B, Berkshire; D, Duroc. Family AY is the Awa-York population and the others are F_2 _families constructed with a Japanese wild boar and two Landrace females (LWb), a Landrace male and two Meishan females (ML), a Meishan male and a Landrace female (LM), a Large White male and a Meishan female (MW), a Large White male and a Jinhua female (JW), a Clawn miniature male and a Berkshire female (BC), a Duroc male and five Jinhua females (JD), and a Japanese wild boar and three Large White females (WWb).

For further analysis, nine microsatellite markers (*SJ7113*, *SJ7139*, *SJ7136*, *SJ7126*, *SJ7121*, *SJ7114*, *SJ7099*, *SJ7107*, and *SJ7103*) were developed from swine genome draft sequences, namely BAC clones CH242-92H3 and CH242-154M12, to which *SJ7106*, *SJ7101*, and *SJ7008 *were assigned (Additional file 2 Table S1); the subsequent analysis revealed that the haplotypes of seven markers, from *SJ7121 *to *SJ7103*, were conserved among the 19 Q alleles of the AY population (upper red box in Figure 3). The two markers *SJ7106 *and *SJ7101 *were located in the conserved region.

### Defining the QTL region by using the parental animals in F_2 _families

**Figure 4:**
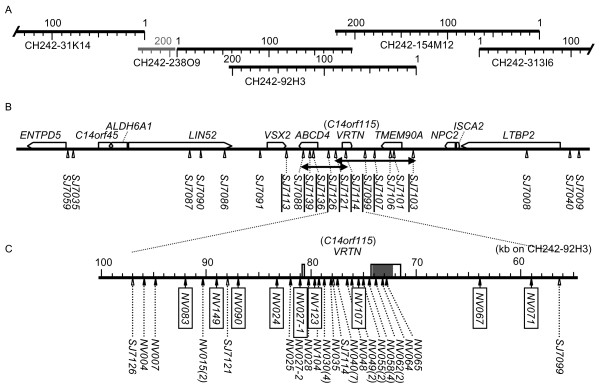
**Genomic structure of the QTL region for number of vertebrae**. A. BAC clones used for swine genome sequencing by the International Swine Genome Sequencing Consortium. B. Gene map and microsatellite markers. In humans, *VRTN *has been submitted to public databases as *C14orf115 *encoding a hypothetical protein. Small triangles indicate positions of microsatellite markers. Double-headed arrows show conserved regions. For the 19 Q alleles of the Large White population (AY population), the haplotypes at seven markers from *SJ7121 *to *SJ7103 *were conserved. For the 14 Q alleles in the F_2 _families, the haplotypes at six markers from *SJ7088 *to *SJ7114 *were conserved. C. The QTL interval defined by haplotype analyses with microsatellite markers. The *VRTN *gene consisted of two exons (boxes) and was located in this interval. The shadowed box is the coding region. White triangles indicate positions of microsatellite markers and black triangles indicate polymorphic sequence-tagged sites (STSs). Numbers in parentheses are numbers of polymorphic sites at each STS. A total of 42 polymorphic sites, which formed two major haplotypes in the AY population, were present. Candidate sites are boxed. Information on polymorphisms is given in Table 3. The sequence of this region was submitted to GenBank/EMBL/DDBJ as AB554652.

Haplotype analysis was performed also with the 11 European-breed pigs used as parents in the F_2 _families; the QTL alleles (14 Q and eight wt alleles) of these parents were characterized in our previous study [6]. For the 14 Q alleles, the haplotypes of six markers from *SJ7088 *to *SJ7114 *were conserved (lower red box in Figure 3). Only two markers (*SJ7121 *and *SJ7114*) were common to these two sets of conserved haplotypes. The QTL was therefore judged to be located between the flanking markers *SJ7126 *and *SJ7099*, which were approximately 41 kb apart (Figure 4).

The corresponding region of the human genome encodes a hypothetical protein, C14orf115. Also, in pigs, parts of a transcript for the hypothetical protein have been submitted as expressed sequence tags (ESTs BE032408.1, BE032606.1, and BF198822.1) and miscRNAs (XR_045716 and XR_045719). We also cloned the corresponding cDNA (AB550854) from swine embryos by RT-PCR (Additional file 4 Figure S2). The swine gene consisted of two exons, as in humans; the start codon, the acceptor and donor sites of the intron, and the stop codon in the second exon were identical to those in humans.

### Association of SNPs with number of vertebrae, and linkage disequilibrium analysis

**Figure 5:**
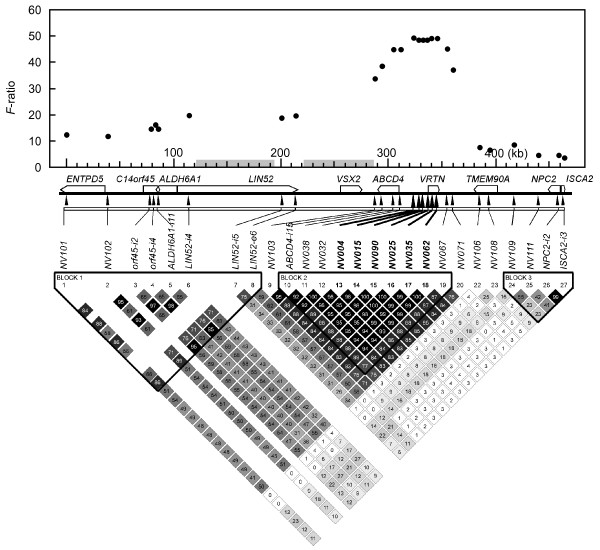
**Association of SNPs with vertebral number, and linkage disequilibrium analysis**. In the 450-kb region around the QTL, SNPs were developed and were genotyped by using 199 independent meat animals, for which the numbers of vertebrae were scored. The associations between SNPs and numbers of vertebrae are presented, with *F*-values. Linkage disequilibrium was analyzed by using Haploview software, and the R^2 ^values are presented in the boxes. For the two gray intervals in the graph, SNPs (minor allele frequency > 0.01) were not detected in our DNA samples from European breed pigs.

By using the sequences of the CH242 series of BAC clones of the SGSC, we analyzed the genomic structure of the 450-kb region around the QTL and constructed a provisional gene map (Figure 4; Additional file 5 Table S3). We also developed 26 single nucleotide polymorphism (SNP) markers (Figure 5; Additional file 6 Table S4) and used them for a linkage disequilibrium analysis in 199 independent meat animals produced by mating Duroc sires and F_1 _(Landrace and Large White) dams. For these meat animals, the number of vertebrae was also scored. Three haplotype blocks were detected, and six SNPs (*NV004*, *NV015*, *NV090*, *NV025*, *NV035*, and *NV062*), which were highly associated with each other (R^2 ^> 0.98), presented the highest associations with the number of vertebrae (Figure 5). These SNPs increased the number of vertebrae with an additive effect of 0.51 and a dominance effect of 0.04 (calculated from Table 2). The change in vertebral number occurred in the thoracic vertebrae; differences in the average number of lumbar vertebrae were not seen among the QTL types (Table 2). The six SNPs, which were between *SJ7126 *and *SJ7099*, were located from the promoter region to the second exon of the gene encoding the hypothetical proteins (Figure 4). These results strongly suggested that this hypothetical protein was responsible for the QTL on SSC7 for number of vertebrae in pigs; we named the encoding gene *vertnin *(*VRTN*).

**Table 2:** Summary of QTL effect in meat animals

	Total number of vertebrae							
					
	20	21	22	23				
	
QTL	Number of individuals				Total	Average		
Q/Q		21	31	2	54	21.65		
Q/wt	3	88	21	1	113	21.18		
wt/wt	14	16	2		32	20.63		

	17	125	54	3	199	21.22		

	No. of thoracic vertebrae				No. of lumbar vertebrae			
				
	14	15	16		5	6	7	
				
QTL	No. of individuals			Average	No. of individuals			Average

Q/Q		12	42	15.78	11	39	4	5.87
Q/wt	1	78	34	15.29	21	84	8	5.89
wt/wt	14	16	2	14.63	6	20	6	6.00

	15	106	78	15.32	38	143	18	5.90

QTL type was judged with *NV090*.

### Polymorphism of the QTL region

Polymorphism analysis of the 41-kb region (AB554652) between *SJ7126 *and *SJ7099*, excluding the PRE1 sequences (swine SINE elements), revealed that only two haplotypes existed in the AY population, and these two haplotypes corresponded to the Q and wt alleles (Table 3). These two haplotypes consisted of 42 polymorphic sites (SNPs and indels), including the six SNPs mentioned above (Figure 4; Table 3; Additional file 6 Table S4).

**Table 3:** Haplotypes of polymorphic sites on number-increase-type and wild-type alleles of swine *VRTN *gene

STS for SNP/indel ^a^	Q (number-increase type)	q (wild type, major)	q' (wild type, minor; found in Landrace)
*NV004*	T	C	T
*NV007*	C	G	C
*NV083*	A	G	G
*NV015(2)*	A, G	C, A	A, G
*NV149*	T	A	A
*NV090*	T	C	C
*NV024*	A	T	T
*NV025*	G	A	G
*NV027(2)*	C, C	A, T	A, C
*NV028*	A	G	A
*NV123*	ins (PRE1 291 bp)	del (PRE1 291 bp)	del (PRE1 291 bp)
*NV104*	G	A	G
*NV030(4)*	A, T, C, T	G, G, T, C	A, T, C, T
*NV035*	C	G	C
*NV040(7)*	del, A, A, G, G, C, T	A, G, T, A, A, T, C	del, A, A, G, G, C, T
*NV048*	A	G	A
*NV049(2)*	T, C	C, T	T, C
*NV107*	AAA	AAAAA	AAAAA
*NV055(2)*	T, GA	C, del	T, TGA
*NV058(4)*	T, T, G, G	C, C, A, C	T, T, G, G
*NV062(2)*	C, G	T, A	C, G
*NV064*	G	A	G
*NV065*	C	T	C
*NV067*	del	C	C
*NV071*	C	A	A

The nine underlined polymorphic sites were segregated between the Q and wt (q and q') alleles.

^a ^Numbers in parentheses are numbers of polymorphic sites at each STS.

We next analyzed the 42 polymorphic sites in the 11 European-breed pigs used as parents of the F_2 _families (14 Q and eight wt alleles). All 14 Q alleles had the same haplotypes as that of the Q allele in the AY population. The same haplotypes (q in Table 3) as those in the AY population were detected in seven of eight wt alleles. In the other wt allele, which was in a Landrace sow, a unique haplotype (q' in Table 3) was detected and 33 of the polymorphic sites were identical to those of the Q allele in the AY population. These 33 were therefore excluded as candidate polymorphic sites. Of the remaining candidate polymorphic sites, five were located in the promoter region, two were in the intron, and two were in the downstream region of the gene (Figure 4). Among the nine polymorphisms, six are SNPs, two are small size of insertion or deletion, and the other is an insertion of a PRE1 element (291 bp) into the intron of the Q allele. These polymorphic sites of *VRTN *are highly related to the heterogeneity of the number of vertebrae in commercial-breed pigs, so this information will be useful for genetic diagnosis in breeding populations.

### Expression of *VRTN *gene in swine embryos

We examined the promoter activities of the swine *VRTN *gene by using mouse ES cells (P19 and CGR8) but found no difference between the Q and wt alleles (data not shown). We detected the transcripts of mouse *VRTN *by RT-PCR at embryonic days 6 to 10.5 (data not shown) and also tried to detect the transcripts of *VRTN *in swine embryos. For this analysis, we prepared heterozygous embryos by mating between Q and wt homozygotes, as judged by the haplotypes of *NV024*, *NV107*, and *NV062*. At five embryonic stages--days 8.0, 8.5, 10.0, 12.0, and 14.0 postcoitus--embryos were collected from the uterus and RT-PCR was performed with RNA from total embryos. Amplifications were detected similarly through these periods. The PCR products were TA-cloned and SNPs were typed by a sequence-based method for each stage (Additional file 7 Figure S3). In two samples at day 8.0, only 1.1% and 3.5% of clones were derived from the wt allele; the majority were from the Q allele (Table 4). By day 8.5, the proportion of clones from the wt allele had increased to 33.0% and 50.3%, and by day 10.0 similar numbers of clones were derived from the Q and wt alleles. By days 12 and 14 all of the clones were from the Q allele. These results showed that regulation of expression of the Q allele changed with the embryonic stage, and that the Q allele was expressed more abundantly than the wt allele.

**Table 4:** Expression of *VRTN *alleles in heterozygous embryos of pigs

	Expression rate (%)	
Embryonic day	Q	wt
8.0 ^a ^sample 1	98.9	1.1
sample 2	96.5	3.5
8.5 ^a ^sample 1	67.0	33.0
sample 2	49.7	50.3
10.0 (3 embryos)	54.5 ± 3.7	45.5 ± 3.7
12.0 (3 embryos)	100.0 ± 0.0	0.0 ± 0.0
14.0 (3 embryos)	100.0 ± 0.0	0.0 ± 0.0

^a ^Each sample contained five embryos.

## Discussion

To map the QTL on SSC7, we used the allele type in the QTL (Q or wt), not the phenotype (vertebral number: 20, 21, 22, or 23). By using half-sib analysis with approximately 1100 progeny animals, we defined the 30 alleles of 15 parent animals. The amount of data seems small, but it is worthwhile because it is qualitative, not quantitative. These 15 animals were from a closed breeding population, the AY population. This population was derived from 10 sires and 65 dams as founder individuals and was bred for seven generations (1987 to 1993), in which average daily gain and backfat thickness were major objectives of breeding but not the number of vertebrae. After 1993, the population was maintained with 10 sires and 35 dams. We think that a large degree of recombination was accumulated in this population, making the population favorable for IBD analysis. On the other hand, unrelated individuals were used as the founders, and we expected that the Q alleles of some of these pigs would have common sequences in a limited region near the QTL, enabling high resolution for fine mapping similar to those used in association studies.

We started fine mapping of the QTL over a region of approximately 9 Mb to give a 95% confidence interval in the QTL analysis. In the first step of haplotype analysis for the 19 Q and 11 wt alleles (Figure 2), the candidate region was fortunately narrowed to approximately 300 kb (between *SJ7088 *and *SJ7040*), but a conserved region contained only two markers in the 19 Q alleles. The second step of the analysis, using densely located markers, revealed the conserved region with seven markers (no more than 200 kb) in the Q alleles of the AY population.

In our previous study [6], we characterized the QTL alleles of parental pigs in the F_2 _families, and we used these animals for further analysis. The 14 Q alleles of these pigs (five Landrace, two Berkshire, two Duroc and five Large White) presented a conserved haplotype from *SJ7088 *to *SJ7114*. As a final mapping result, the candidate region for the QTL was an approximately 41-kb region between *SJ7126 *and *SJ7099*, which included the common haplotype within all the Q alleles in the present study.

The result of the fine mapping was confirmed by linkage disequilibrium analysis and an association study in independent meat animals produced with Duroc sires and F_1 _(Landrace × Large White) dams. The 41-kb region was included in a haplotype block, and the SNPs developed in this region were highly associated with the number of vertebrae. We therefore concluded that the QTL was located in the 41-kb region.

In the 41-kb region, transcripts were detected in pigs as EST in the embryonic stages. They encoded parts of a putative uncharacterized protein, which is not a member of any other known gene family. We cloned the cDNA covering the putative open reading frame from the swine embryos and we named it *vertnin *(*VRTN*) as the gene responsible for the QTL for vertebral number in pigs. A domain search of the swine homolog (C14orf115) in Ensemble (http://uswest.ensembl.org/index.html) revealed that it had a similar motif to the helix-turn-helix domain of Transposase IS3/IS911 [16]. Although this domain is reported to be unique to bacteria, vertnin is expected to have DNA-binding activities. In our preliminary study the green fluorescent protein (GFP)-fused vertnin protein was expressed in the nuclei of cultured cells (Additional file 8 Figure S4). The QTL on SSC7 in this study affected the number of thoracic vertebrae but not the number of lumbar vertebrae, whereas the QTL on SSC1 affected the numbers of both thoracic and lumbar vertebrae [6]. This evidence suggested that the number-increase allele of swine *VRTN *added an additional thoracic segment in the animal. We suspect that the expression pattern of *Hox *genes (e.g. *Hoxa-9 *or *Hoxc-9*, which are expressed in the terminal region of the thoracic segment and more posterior portion of mouse embryos; [17,18]) could be altered. A series of genome sequencing projects (funded by National Human Genome Research Institute; http://genome.gov/) suggested that orthologs exist not only in other mammals but also in birds and fish, and encode conserved proteins (Additional file 9 Figure S5). Vertnin is therefore likely to be an essential factor for development of the embryo in a wide range of organisms. Its functional analysis may provide novel findings in the areas of developmental biology (e.g., somitogenesis or morphogenesis), so we are planning to perform a functional analysis with model organisms such as the mouse or chicken.

Polymorphism analysis of the 41-kb region showed that the AY population had only two haplotypes (Q and q), which consisted of 42 polymorphic sites. Another haplotype (q') was detected in the wt allele of a Landrace sow, in which the q' allele had no significant effect (calculated as 0.05) and the other allele (Q) increased 0.55 of vertebral number [6]. In our preliminary study it was also found in the sequence of the BAC clone L261J7 [19] (Additional file 2 Table S1). There were nine candidate polymorphic sites, and they were highly associated with each other in European commercial-breed pigs. We cannot define the causative polymorphic sites genetically, but these nine sites were associated with the number of vertebrae in commercial-breed pigs, and this information will be useful for genetic diagnosis in breeding populations.

In the swine *VRTN *gene, we found one nonsynonymous substitution, *NV064 *(G→A at nucleotide 1247 of AB550854; Gly365Asp), but it was excluded from the candidate sites because the same nucleotide G was located in the Q and q' alleles (Table 3). We detected a change in the transcription of *VRTN *with changes in embryonic stage. It is possible that this change in expression with stage is the origin of the QTL. Among the polymorphisms of the swine *VRTN *gene, an insertion of a PRE1 element (one of the swine SINE elements) into the intron of the Q allele, along with the SNPs in the promoter region, may be a cause of the changes in expression of this allele with embryonic stage. In mouse organogenesis, expression of some genes is regulated by a mechanism that includes the activation of a SINE B2 repeat [20]. In this case, the SINE B2 acts as a boundary between the heterochromatin and euchromatin, partly by the activation of pol III and pol II RNA polymerase. In another case, SINE elements have been reported to act as enhancer elements, as is the case with the *FGF8 *gene [21]. In the swine *VRTN *gene, transcripts around the PRE1 element were detected on the Q allele by RT-PCR in our preliminary study (data not shown). To further explore the causative mutation as well as the biological changes induced by it, we are using molecular biological techniques to study the regulation of swine *VRTN *expression.

## Conclusions

Genetic diversity of *VRTN *is the suspected cause of the heterogeneity of number of vertebrae in commercial-breed pigs associated with SSC7, so the polymorphism information should be directly useful for assessing the genetic ability of individual animals. We expect that our identification of the *VRTN *gene will be of great benefit to the pork production industry. The number-increase allele of swine *VRTN *affected the vertebral formula, especially in the thoracic segment. The functional analysis of *VRTN *may provide novel findings in the areas of developmental biology.

## Methods

### Animals

DNA samples and data for number of vertebrae came from a Large White population (named Awa-York, AY) at the Livestock Research Institute of Tokushima Prefecture in Japan. ("Awa" is the old name for Tokushima Prefecture.) This population was derived from 10 sires and 65 dams as founder individuals and was bred as a closed population for seven generations (1987 to 1993), with eight to 11 sires and 28 to 36 dams. The population was then maintained with 10 sires and 35 dams. The population in this study contained some individuals that had been used in our previous study [6].

We also used DNA samples from European-breed parent pigs of the F_2 _families from our previous study [6]. These families were produced by crossing a Landrace male with two Meishan females (ML), a Japanese wild boar with two Landrace females (LWb), a Large White male with a Jinhua female (JW), a Clawn miniature [22] male with a Berkshire female (BC), a Large White male with a Meishan female (MW), a Meishan male with a Landrace female (LM), a Duroc male with five Jinhua females (JD), and a Japanese wild boar with three Large White females derived from the Awa-York population (WWb; [23]).

DNA samples from the 199 meat animals independently obtained from Chiba Prefecture, Japan, were prepared from the skin after slaughter; the numbers of vertebrae were also scored. These meat animals had been produced by mating Duroc sires to F_1 _dams of Landrace and Large White.

### Scanning for QTL on SSC7 for number of vertebrae

A QTL scan on SSC7 was performed for number of vertebrae in the Jinhua × Duroc cross population (JD family). An interval mapping based on the least-squares method developed for an outbred population [24] was used. The 95% confidence intervals for the QTL on SSC7 were obtained by bootstrap analysis of 10,000 repetitions with QTL Express software [25,26]. In this analysis, a linkage map constructed with the JD family was used.

### Isolation of bacterial artificial chromosome (BAC) clones and development of microsatellite markers

A comparative map between pig and human [13] showed that the QTL region of SSC7 corresponds to a region on human chromosome 14. Swine sequence tagged sites (STSs) were developed from swine ESTs or swine genomic sequences, which were obtained by BLAST searches with human gene sequences (Additional file 2 Table S1). BAC clones [19] were screened with these swine STSs, and microsatellite sequences were isolated from the BAC clones by using a direct sequencing method reported previously [27]. Recently, the SGSC has made progress in swine genome sequencing [14]. The sequences of the microsatellite markers were assigned in the swine genome draft sequence, Sscrofa9 assembly as well as the BAC clones (CH242-serieas) for the sequencing.

### Half-sib analysis on SSC7 for number of vertebrae in a Large White population

We scored the number of vertebrae in 1338 individuals produced from a Large White population (AY population). The numbers of vertebrae were 20 (<1%), 21 (33%), 22 (64%), and 23 (3%). Using the microsatellite markers *SW147 *(90.1 cM on the MARC map; [28]), *SW252 *(99.4 cM), and *S0115 *(102.2 cM), which were located on SSC7 near the QTL for number of vertebrae, we genotyped the 1338 progeny as well as their 19 sires and 69 dams. Haplotypes consisting of these three microsatellite markers on each homologous chromosome were reconstructed by using the genotype data of the sires, the dams, and their progeny. Among the 1338 progeny, 1122 were classified into two groups on the basis of the haplotype inherited from their sire or dam. We compared the average numbers of vertebrae in the two groups of progeny for each sire or each dam and used *t*-tests to evaluate the significance of the differences. Sires and dams were considered to be heterozygous (Q/wt) at the QTL when significant differences (P < 0.01) were detected. Moreover, to detect sires and dams homozygous for the QTL, we performed *Z*-tests in accordance with the method of Nezer *et al*. [15]. The Q-to-wt substitution effect was set at 0.49, which was derived from the value 0.49 ± 0.10 calculated from nine heterozygotes in the AY population. Sires and dams were judged to be homozygous when *Z *< -2.0. QTL types of homozygotes (Q/Q or wt/wt) were judged by Tukey's multiple comparison of the average number of vertebrae of each progeny group with those (Q: 21.92 and wt: 21.43) of the total groups of Q and wt alleles inherited by the progeny (371 and 382, respectively). QTL types of homozygotes were confirmed by comparison of the haplotypes of 57 markers in the 95% confidence interval with those of heterozygotes (Table 1). Reconstruction of the haplotypes of the 57 markers is described below.

### Haplotype analysis of high-density microsatellite markers in the 95% confidence interval of the QTL for number of vertebrae on SSC7

For the sires and dams of the AY population and for the parent pigs of the F_2 _families, haplotypes of 57 microsatellite markers in the 95% confidence interval (approximately 5 cM, 9 Mb) of the QTL on SSC7 were analyzed. Haplotypes of the markers were reconstructed by using genotype data on the sires, dams, and their progeny: samples of one sire, one dam, and six progeny were used for each full-sib family. For IBD identification, the haplotypes of their progenitor animals were determined and recombination sites were analyzed. IBD regions supported by more than four markers were shown in Figures 2 and 3.

### Structure analysis of the swine genomic region around the QTL for number of vertebrae

In the QTL region on SSC7, we found some errors in the Sscrofa9 assembly. The sequence of BAC clone CH242-92H3 (Figure 4) was inserted inversely between those of CH242-238O9 and CH242-154M12 without overlapped sequences, and some regions were duplicated (*SJ7088*, *SJ7106*, *SJ7101*, *SJ7008 *in additional file 2 Table S1). Then we constructed the gene map again. With the sequences of the BAC clones CH242-31K24, CH242-238O9, CH242-92H3, CH242-154M12, and CH242-313I6, BLAST searches were performed against the human genome sequence. Sequence information on the genes in the human homologous region was used for the construction of a provisional gene map (Additional file 5 Table S3). In each gene, exons were estimated with the human gene sequence or swine mRNA sequences. The gt-ag rule was confirmed for each intron. For the swine *VRTN *gene, RT-PCR was performed with three sets of primer pairs-sVRTN 1, 5'-agacggtccatgctcaatg-3' and 5'-tgcagtgctccaggtacaac-3'; sVRTN 2, 5'-aggaggtggaggctgaaagt-3' and 5'-actcaggtccctgaccctct-3'; and sVRTN 3, 5'-cagcttctgttggggaaaag-3' and 5'-ctgtggggtccagaacagat-3'-in the genomic sequence corresponding to the human *C14orf115 *sequence (Additional file 4 Figure S2).

### Association of SNPs with number of vertebrae, and linkage disequilibrium analysis around the QTL region

PCR primers were designed on the basis of the sequences of the BAC clones of the SGSC, and SNPs were developed from eight samples of DNA from meat animals produced by the mating of Duroc sires and F_1 _dams of Landrace and Large White (Additional file 6 Table S4; Figure 5). SNPs were genotyped for 199 independent samples from meat animals, for which the numbers of vertebrae were scored. Analysis of variance was performed for each SNP. Linkage disequilibrium was analyzed in the same 199 meat animals by using Haploview software [29]; the R^2 ^values are shown in Figure 5.

### Polymorphism analysis of swine *VRTN *gene

Polymorphism analysis for the approximately 41-kb region between microsatellite markers *SJ7126 *and *SJ7099 *was performed in the AY population. DNA samples from sires 3605 (Q/Q), 5002 (Q/Q), 1606 (Q/wt), and 5901 (Q/wt), and from dam 2206 (wt/wt) of the AY population (Table 1) were used. Sequences were determined by application of a series of PCR and direct sequencing methods to each sample. Polymorphic sites segregating between the Q and wt alleles, except for those in PRE1 elements, are shown in Figure 4 and Table 3. These polymorphic sites were elucidated by using other DNA samples from the AY population and the parent pigs of the F_2 _families, for which QTL types were determined. A haplotype that we found in a Landrace female and considered to be a wt allele is also presented in Table 3.

### Expression analysis of *VRTN *in swine embryos

Heterozygous embryos were prepared by reciprocal matings of Q and wt homozygotes, which were determined by the haplotypes of *NV024*, *NV107*, and *NV062 *in the swine *VRTN *gene. At five embryonic stages--days 8.0, 8.5, 10.0, 12.0, and 14.0 postcoitus--embryos were collected from the uterus after slaughter. RNA was prepared with Trizol (Invitrogen) and RT-PCR was performed with PCR primers for *NV062 *(Additional file 7 Figure S3). Amplified DNA fragments were cloned by using a TOPO-TA cloning kit (Invitrogen), and two SNPs in *NV062*, which was located in the second exon, were genotyped from 96 clones in each stage by direct-sequencing of PCR fragments from the inserts. On each of days 8.0 and 8.5, five embryos were taken together as one sample and RNA was prepared. At the other stages postcoitus, RNA was prepared separately from each embryo. At each of days 8.0 and 8.5, two samples were from reciprocal matings. At day 10, one embryo was from a cross between a wt/wt male and a Q/Q female, and two were from a reciprocal cross. At day 12, embryos were from a cross between a wt/wt male and a Q/Q female. At day 14, embryos were from a cross between a Q/Q male and a wt/wt female.

## Authors' contributions

SM designed the experiment, coordinated IBD and LD mapping, analyzed genome structure, performed association study, coordinated expression analysis of swine embryos, and wrote the manuscript. SS developed STSs, microsatellite markers and SNPs, coordinated IBD and LD mapping, and performed polymorphism analysis. MN collected samples and phenotypes for IBD analysis. TM performed expression analysis of GFP-fused proteins, and coordinate expression analysis of swine embryos. GY and NI collected and prepared the samples of swine embryos. TY collected and prepared the samples for LD mapping and association study. TH performed QTL mapping and statistical analysis. TA coordinated expression analysis of swine embryos, was involved in discussions about all of the analyses performed, and assisted in manuscript preparation. All authors reviewed the manuscript.

## Supplementary Material

Additional file 1 **Figure S1: Development of microsatellite markers in the 95% confidence interval of the QTL on SSC7**. **A. **A part of the gene map for the human chromosome 14 (from the human genome reference genome assembly of NCBI, Build 37.1). Human sequences in the region corresponding to the QTL on SSC7 were used to search for homologous swine sequences by BLAST analysis. PCR primers for STSs were designed in these swine sequences. **B**. Swine BAC clones screened with STSs. **C**. Swine microsatellite markers developed in this study. Microsatellite markers were isolated from the BAC clones by a direct sequencing method using two-nucleotide repeats such as (CA)_10 _for sequencing primers. **D**. A part of the SSC7 sequence map (from the swine genome draft sequence, Sscrofa9 assembly, published by the International Swine Genome Sequencing Consortium). The microsatellite markers developed in this study and those on a linkage map developed by Rohrer [28] were assigned to the Sscrofa9 assembly. The underlined markers have not yet been found in the Sscrofa9 assembly. Dotted lines indicate that markers were assigned to multiple positions. **E**. A part of the SSC7 linkage map for microsatellite markers, developed by Rohrer [28]. Click here for file

Additional file 2 **Table S1: Development of swine STSs and microsatellite markers**. Click here for file

Additional file 3 **Table S2: Information on microsatellite markers**. Click here for file

Additional file 4 **Figure S2: Swine *VRTN *cDNA**. A swine *VRTN *cDNA. (number-increase-type; AB550854) was cloned by RT-PCR with three primer pairs: sVRTN 1 (red underlines), sVRTN 2 (green), and sVRTN 3 (blue). Comparison with the swine genome draft sequences revealed that exon 1 extended from the 1st to the 152nd nucleotides and exon 2 ranged from the 153rd to the end. The coding region was 2,094 bp long (154-2,247) and predicted to encode a protein of 698 amino acids. The positions of SNPs in a Large White population (the AY population) are shown with blue background. Click here for file

Additional file 5 **Table S3: Locations of genes surrounding the QTL**. Click here for file

Additional file 6 **Table S4: Polymorphic markers used in this study**. Click here for file

Additional file 7 **Figure S3: Expression analysis of *VRTN *in swine embryos**. Click here for file

Additional file 8 **Figure S4: Expression of the green fluorescent protein (GFP)-fused vertnin in cultured cells**. The plasmid vector pcDNA-DEST47-VRTN, which encoded GFP-fused vertnin, was constructed from *VRTN *cDNA and pcDNA-DEST47 plasmid vector (Invitrogen). NIH-3T3 and HeLa cells (1 × 10^4 ^cells/chamber) were seeded on BioCoat Poly-D-Lysine 4-well Culture Slides (BD Biosciences), and then the plasmid vectors pcDNA-DEST47-VRTN and pcDNA/GW-47/CAT (Invitrogen), which encoded a GFP-fused CAT (chloramphenicol acetyltransferase) and was a control for cytoplasmic expression, were transfected into cells by using FuGENE 6 (Roche Diagnostics). Forty-eight hours after transfection, the cells were washed and the nuclei were counterstained with 4',6-diamidino-2-phenylindole (DAPI) (Invitrogen). The cells were mounted with the anti-bleaching reagent DABCO (Invitrogen) and analyzed by fluorescence microscopy to examine green (GFP) and blue (DAPI) fluorescence. DAPI staining indicates the locations of nuclei, and GFP-fused vertnin has a similar expression pattern in both types of cell. Click here for file

Additional file 9 **Figure S5: Orthologs of swine vertnin protein. A**. In the public database, protein sequences probably coded by orthologous genes of swine *VRTN *were found not only in mammals, including opossum, but also in a bird (zebra finch) and fish (zebrafish and tetraodon). The orthologous genes were conserved at the start and stop codons, so it seems that *VRTN *encodes a functional protein. Alignment was performed with ClustalW software. Underlines in the swine sequence indicate the homologous region to helix-turn-helix domain of Transposase IS3/IS911. **B**. The identities (%) of amino acid sequences to swine VRTN. **C**. Phylogenic tree of vertnin proteins constructed with ClustalW software. Click here for file
