# Supplementary figures and images for: Identification of a second gene associated with variation in vertebral number in domestic pigs
Source: BMC Genet. 2011 Jan 14;12:5. doi: 10.1186/1471-2156-12-5 (PMC3024977; doi:10.1186/1471-2156-12-5)

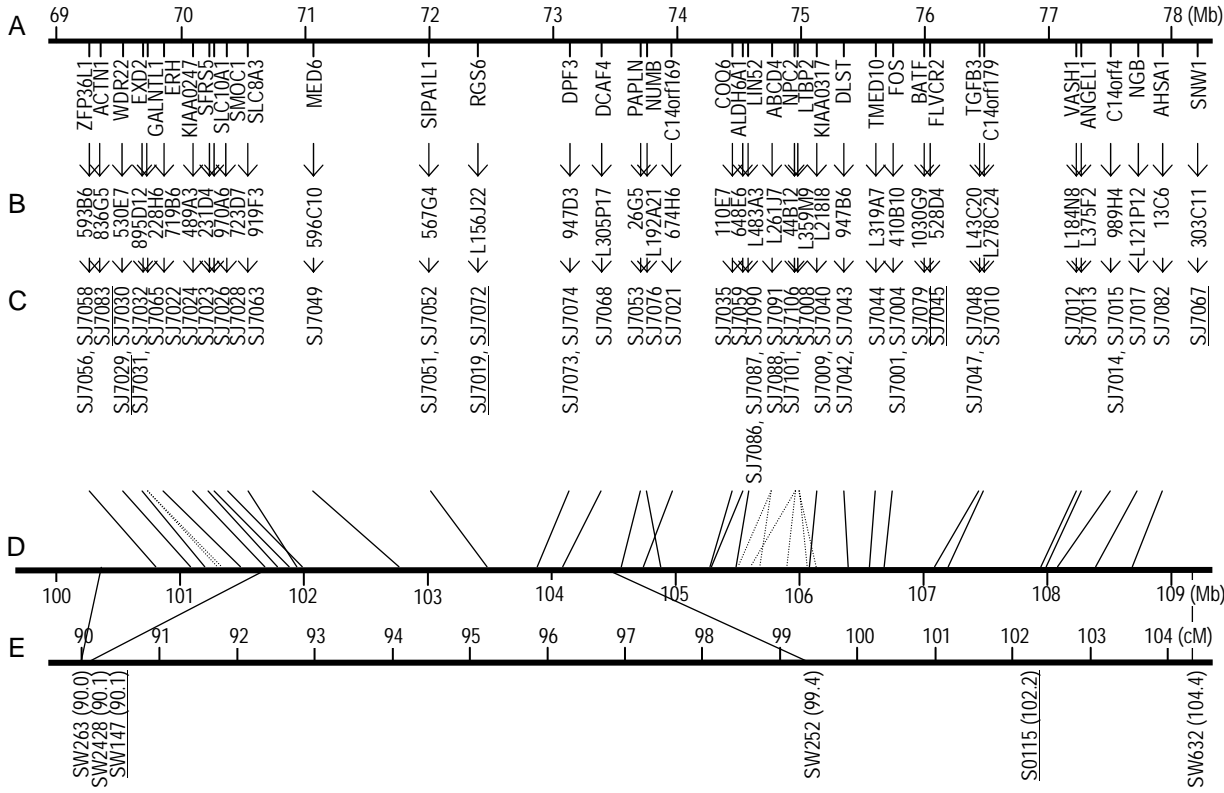

Supplement: Additional file 1 — Figure S1: Development of microsatellite markers in the 95% confidence interval of the QTL on SSC7. A. A part of the gene map for the human chromosome 14 (from the human genome reference genome assembly of NCBI, Build 37.1). Human sequences in the region corresponding to the QTL on SSC7 were used to search for homologous swine sequences by BLAST analysis. PCR primers for STSs were designed in these swine sequences. B. Swine BAC clones screened with STSs. C. Swine microsatellite markers developed in this study. Microsatellite markers were isolated from the BAC clones by a direct sequencing method using two-nucleotide repeats such as (CA)10 for sequencing primers. D. A part of the SSC7 sequence map (from the swine genome draft sequence, Sscrofa9 assembly, published by the International Swine Genome Sequencing Consortium). The microsatellite markers developed in this study and those on a linkage map developed by Rohrer [28] were assigned to the Sscrofa9 assembly. The underlined markers have not yet been found in the Sscrofa9 assembly. Dotted lines indicate that markers were assigned to multiple positions. E. A part of the SSC7 linkage map for microsatellite markers, developed by Rohrer [28]. [file 1471-2156-12-5-S1.PDF]

Embryo (Q/wt)

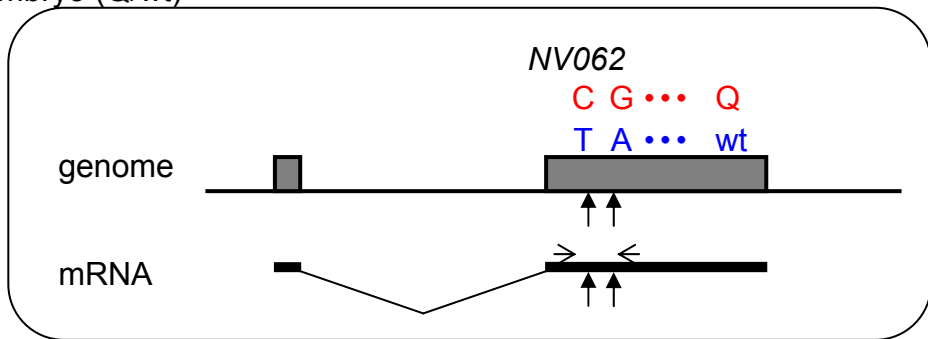

RT-PCR (NV062)

TA-cloning

Sequence-based typing

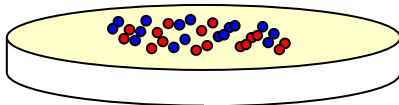

Supplement: Additional file 7 — Figure S3: Expression analysis of VRTN in swine embryos. [file 1471-2156-12-5-S7.PDF]

# VRTN-GFP

DAPI

GFP

NIH-3T3

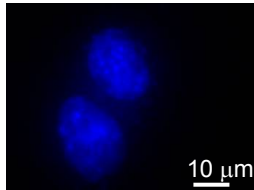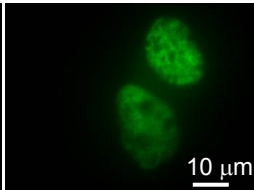

HeLa

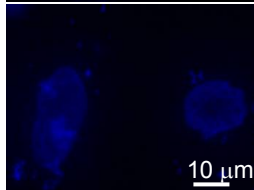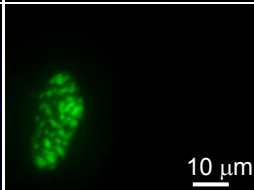

# CAT-GFP

DAPI

GFP

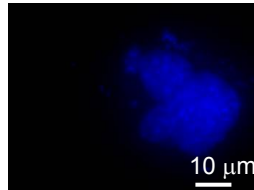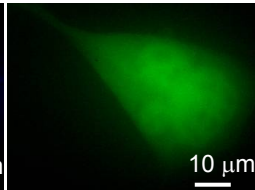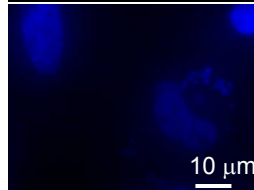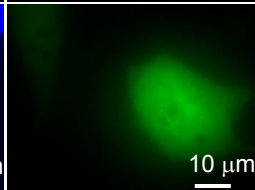

Supplement: Additional file 8 — Figure S4: Expression of the green fluorescent protein (GFP)-fused vertnin in cultured cells. The plasmid vector pcDNA-DEST47-VRTN, which encoded GFP-fused vertnin, was constructed from VRTN cDNA and pcDNA-DEST47 plasmid vector (Invitrogen). NIH-3T3 and HeLa cells (1 × 10^4^ cells/chamber) were seeded on BioCoat Poly-D-Lysine 4-well Culture Slides (BD Biosciences), and then the plasmid vectors pcDNA-DEST47-VRTN and pcDNA/GW-47/CAT (Invitrogen), which encoded a GFP-fused CAT (chloramphenicol acetyltransferase) and was a control for cytoplasmic expression, were transfected into cells by using FuGENE 6 (Roche Diagnostics). Forty-eight hours after transfection, the cells were washed and the nuclei were counterstained with 4',6-diamidino-2-phenylindole (DAPI) (Invitrogen). The cells were mounted with the anti-bleaching reagent DABCO (Invitrogen) and analyzed by fluorescence microscopy to examine green (GFP) and blue (DAPI) fluorescence. DAPI staining indicates the locations of nuclei, and GFP-fused vertnin has a similar expression pattern in both types of cell. [file 1471-2156-12-5-S8.PDF]
