# Supplementary material for: Identification of a second gene associated with variation in vertebral number in domestic pigs
Source: BMC Genet. 2011 Jan 14;12:5. doi: 10.1186/1471-2156-12-5 (PMC3024977; doi:10.1186/1471-2156-12-5)
Supplement: Additional file 4 — Figure S2: Swine VRTN cDNA. A swine VRTN cDNA. (number-increase-type; AB550854) was cloned by RT-PCR with three primer pairs: sVRTN 1 (red underlines), sVRTN 2 (green), and sVRTN 3 (blue). Comparison with the swine genome draft sequences revealed that exon 1 extended from the 1st to the 152nd nucleotides and exon 2 ranged from the 153rd to the end. The coding region was 2,094 bp long (154-2,247) and predicted to encode a protein of 698 amino acids. The positions of SNPs in a Large White population (the AY population) are shown with blue background. [file 1471-2156-12-5-S4.PDF]

```
agacggtccatgctcaatggtccaccacagatatgaaaccacttctggagtgagagtggcgctcagttgctgcccacagggtgacttaaa   90
tgtcccaagctggaaggtggagagagacgtggacgccccctgggctctgggccaccctcgaggATGACATCTCGGGAGCAGCTGGTGCTG   180
                                                               MetThrSerArgGluGlnLeuValLeu
CAGGTGCTGCAGGAGCTGCAGGAGGCAGTGGAGTCCGAGGGCCTGGAGGGTCTTGTGGGTGCCGCTCTGGAAGCCAAGCAGGTTTTGTCT   270
GlnValLeuGlnGluLeuGlnGluAlaValGluSerGluGlyLeuGluGlyLeuValGlyAlaAlaLeuGluAlaLysGlnValLeuSer
TCCTTTGCTCTCCCCACCTGCCGGGAGGGAGGCCCCGGCCCCCAGGTGCTGGAGGTGGACTCAGTGGCCCTGAGCCTGTACCCAGAGGAT   360
SerPheAlaLeuProThrCysArgGluGlyGlyProGlyProGlnValLeuGluValAspSerValAlaLeuSerLeuTyrProGluAsp
GCTCCCCGGAACATGTTGCCGCTGGTGTGCAAGGGCGAGGGCAGCCTGCTGTTCGAGGCGGCCAGCATGCTGCTCTGGGGTGACTCGGGC   450
AlaProArgAsnMetLeuProLeuValCysLysGlyGluGlySerLeuLeuPheGluAlaAlaSerMetLeuLeuTrpGlyAspSerGly
CTCAGCCTGGAGCTGCGGGCCCGCACGGTGGTGGAGATGCTGCTGCACCGGCACTACTACCTCCAGGGCATGATCGACTCCAAGGTGATG   540
LeuSerLeuGluLeuArgAlaArgThrValValGluMetLeuLeuHisArgHisTyrTyrLeuGlnGlyMetIleAspSerLysValMet
CTGCAGGCTGTGCGCTATTCCCTCTGCTCCGAGGAGTCCCCTGAGATGACCAGCCTGCCCTCCGCCACGCTGGAGGCCATCTTCGACGCG   630   NV062-1 C/T
LeuGlnAlaValArgTyrSerLeuCysSerGluGluSerProGluMetThrSerLeuProSerAlaThrLeuGluAlaIlePheAspAla         NV062-2 G/A
GACGTCAAGGCCACCTGCTTTCCTAGCAGCTTCTCCAATGTGTGGCACTTGTATGCCCTGGCCTCCGTCCTTCAGCGCAACATCTACTCC   720
AspValLysAlaThrCysPheProSerSerPheSerAsnValTrpHisLeuTyrAlaLeuAlaSerValLeuGlnArgAsnIleTyrSer
ATCTACCCCATGCGCAACCTCAAGATCCGGCCCTACTTTAACCGTGTCATCCGGCCCCGCCGCTGTGACCACATGCCCGCCACGCTGCAC   810
IleTyrProMetArgAsnLeuLysIleArgProTyrPheAsnArgValIleArgProArgArgCysAspHisMetProAlaThrLeuHis
ATCATGTGGGCTGGCCAGCCCCTCACCAACCACCTCTTCCGCCACCAGTACTTTGCCCCCGTGGTGGGGCTGGAGGAGGTGGAGGCTGAA   900
IleMetTrpAlaGlyGlnProLeuThrAsnHisLeuPheArgHisGlnTyrPheAlaProValValGlyLeuGluGluValGluAlaGlu
AGTGCCACCACCAGCCTGGCCCCGACGCCTCCAGCCTTGGCCCCGCTGCCCCCGCCCGCCAAGACCCTGGAGCTGCTCAGCCAGGACCCT   990
SerAlaThrThrSerLeuAlaProThrProProAlaLeuAlaProLeuProProProAlaLysThrLeuGluLeuLeuSerGlnAspPro
GGCCTCAGCTACTCCTACCTCTGTGAGCGCTACAGTGTCACCAAGAGCACCTTCTACCGCTGGCGGCGGCAGTCCCAGGAGCACCGGCAG   1080
GlyLeuSerTyrSerTyrLeuCysGluArgTyrSerValThrLysSerThrPheTyrArgTrpArgArgGlnSerGlnGluHisArgGln
AAGGTGGCCACCCGCTTCTCGGCCAAGCACTTCCTGCAGGACAGCTTCCACCGTGGGGGCGTCGTGCCGCTGCAGCAGTTCCTCCAGAGG   1170
LysValAlaThrArgPheSerAlaLysHisPheLeuGlnAspSerPheHisArgGlyGlyValValProLeuGlnGlnPheLeuGlnArg
TTTCCTGAGATCTCCCGCTCTACCTATTATGCCTGGAAGCACGAGCTCTTGGGGTCTGGCACGTGCCAGGCACTAGGCCCGATGGAGGAG   1260   NV064 G/A
PheProGluIleSerArgSerThrTyrTyrAlaTrpLysHisGluLeuLeuGlySerGlyThrCysGlnAlaLeuGlyProMetGluGlu           Gly/Asp
CTGGAGAAGTTGACGGAGGAGCAGGTTGCCGAGGGGCTAGGATGCTCCTCCCCGGCCGTGTCGAGCCCCGGAATGGTCTTGATGCAGCGG   1350
LeuGluLysLeuThrGluGluGlnValAlaGluGlyLeuGlyCysSerSerProAlaValSerSerProGlyMetValLeuMetGlnArg
GCCAAGTTGTACCTGGAGCACTGCATCTCCCTGAATACACTGGTACCCTATCGCTGCTTCAAACGCAGGTTCCCCGGCATCTCCCGGTCC   1440
AlaLysLeuTyrLeuGluHisCysIleSerLeuAsnThrLeuValProTyrArgCysPheLysArgArgPheProGlyIleSerArgSer
ACCTACTACAACTGGCGCCGGAAGGCTCTCCGAAGGAACCCCAGCTTCAAGCCGGCCCCTGTCCTCTCGGTGGCTGGGGCTACCCAGCCA   1530
ThrTyrTyrAsnTrpArgArgLysAlaLeuArgArgAsnProSerPheLysProAlaProValLeuSerValAlaGlyAlaThrGlnPro
GCTTCTGTTGGGGAAAAGGCCTTGCTCCCTTGGGAGGGTGAGGTGGGAGAGGAGGCAGGGAAAGCAACGGGTGGGGGGCAACCTGCCCCG   1620
AlaSerValGlyGluLysAlaLeuLeuProTrpGluGlyGluValGlyGluGluAlaGlyLysAlaThrGlyGlyGlyGlnProAlaPro
CGGGAGTTCCTGCCCCTGAGGATGCCCCTGTCCCGTTGGCAGAGGCGTCTGCGCAGGGAAGCCCGCAAGCAGGTGCTCAGTGGGCACCTC   1710   NV065 C/T
ArgGluPheLeuProLeuArgMetProLeuSerArgTrpGlnArgArgLeuArgArgGluAlaArgLysGlnValLeuSerGlyHisLeu
CCCTTCTGTCGCTTCCGCCTCCGCTACCCAAGCCTGTCACCCTCCACCTTTTGGGTGTGGAAGAGTCTTGCACGGGGCTGGCCCAGAAGC   1800
ProPheCysArgPheArgLeuArgTyrProSerLeuSerProSerThrPheTrpValTrpLysSerLeuAlaArgGlyTrpProArgSer
CTGTCCAAGCTCCATATTCAGGCCCCCACCTTGGGCAAAGGGGGCATTCAGGAGGTGGAGGAGAAACAGGAGAAAGAAGCTGGTAGGAAC   1890
LeuSerLysLeuHisIleGlnAlaProThrLeuGlyLysGlyGlyIleGlnGluValGluGluLysGlnGluLysGluAlaGlyArgAsn
GTGACAGCTGCCATGGCTCCGCCTGCAGGGACCCTGCAGATGACAGCTTCCCCAGGAGAGGATCCAGGGGCGGCCCTGGGAGGGCCTTCC   1980
ValThrAlaAlaMetAlaProProAlaGlyThrLeuGlnMetThrAlaSerProGlyGluAspProGlyAlaAlaLeuGlyGlyProSer
AGAGAGGGGGCCCTACAAGAGGGGGCCATGGCCCAGGGCCGCCCCATGGTGGCAGCGGCAGGTGGCAGGGATGGCCAGGTGCTGGTGATG   2070
ArgGluGlyAlaLeuGlnGluGlyAlaMetAlaGlnGlyArgProMetValAlaAlaAlaGlyGlyArgAspGlyGlnValLeuValMet
GACATGCTCGCCACCACAAAGTTCAAGGCCCAGGCCAAGCTGTTCCTGCAGAAGCGCTTCCAATCCAAGAGCTTCCCCTCCTACAAGGAG   2160
AspMetLeuAlaThrThrLysPheLysAlaGlnAlaLysLeuPheLeuGlnLysArgPheGlnSerLysSerPheProSerTyrLysGlu
TTCAGCGCCCTCTTCCCCCTCACTGCTCGCTCTACCTACTACATGTGGAAGCGCGCCCTGTACGATGGCCTCACCCTGGTCGATGGCTGA   2250
PheSerAlaLeuPheProLeuThrAlaArgSerThrTyrTyrMetTrpLysArgAlaLeuTyrAspGlyLeuThrLeuValAspGly***
cagggcggggtgaaaagggctgggacgaagaaggaggaccgctttggagagggtcagggacctgagttgcccccgcctccccctggcttg   2340
gccaggatgccctttgttcccacaatgtctaccctgaatccaggggcagatttgcattgtttcctagctccagggcagagggagccagag   2430
attagccatctggcctcctgcagagagctacaggaccagtcattctcttcagttgtttaccattcttatttttattatcgtcttagtttt   2520
ttgttttgatgtaaatggctcggccaggccccttgctggtgttggaatccgttatgtgtttggggatccaaaggggaggtggttagcta   2610
aagaatctttcagcgtttcctggggactaagggagtgtcatagcatggccctctgtccccctgggcagaagataggtcttcagttccttt   2700
tggggggttggcaccctccttactccaagatgtttcccaaaggaacattggaaatcagatctggagcaggtcatggcttctgcctggtgcc   2790
cccagggagagggtgccaggcttagggtgagcagaagtggcgatgttaccagaagaaccaaggttggcctttggaaggtcctgagctaga   2880
tcacggaggctggcctgatccttctgaggagtttggtaaggggctgtaggaaagggccctgggtctctgcttgctagagcctggcccatc   2970
tgttctggaccccacag                                                                          2987
```
