# Supplementary material for: Identification of a second gene associated with variation in vertebral number in domestic pigs
Source: BMC Genet. 2011 Jan 14;12:5. doi: 10.1186/1471-2156-12-5 (PMC3024977; doi:10.1186/1471-2156-12-5)

A

```
                      10        20        30        40        50        60        70        80        90       100
             ....|....|....|....|....|....|....|....|....|....|....|....|....|....|....|....|....|....|....|....|
Pig          MTSREQLVLQVLQELQEAVESEGLEGLVGAALEAKQVLSSFALPTCREGGPGPQVLEVDSVALSLYPEDAPRNMLPLVCKGEGSLLFEAASMLLWGDSGL
Cow          MTSREQLVQHVLQELQEAVESEGLEGLVGAALEAKQVLSSFALPTRRGGGPGPQVLEVDSVALSLYPEDAPRNMLPLVCQGEGSLLFEAASLLLWGDAGL
Horse        MKSREQLVQQVLQELQEAVESEGLEGLVGAALEAKQVLSSFTLPTCREGGPGPQVLEVDPVALSLYPEDAPRNMLPLMCKGEGSLLFEAVSMLLWGDAGL
Dog          MTSREQLIQQVLQELQEAVESEGLEGLVSAALEAKQVLSSFTLPSSREGGSSPQVLEVDSVALSLYPEDAPRNMLPLVCKGEGSLLFEAASVLLWGDVGF
Human        MTSRNQLVQKVLQELQEAVECEGLEGLIGASLEAKQVLSSFTLPTCREGGPGLQVLEVDSVALSLYPEDAPRNMLPLVCKGEGSLLFEAASMLLWGDAGL
Rhesus       MTSRNQLVQRVLQELQEAVECEGLEGLVGAALEAKQVLSSFTLPTCREGGPGLQVLEVDSVALSLYPDDAPRNMLPLVCKGEGSLLFEAASMLLWGDAGL
Mouse        MTSRDQLVQQVLRDLQEAVESEGLEGLIGAALEAKQVLSSFTLPICQKGGPGAQVLEVDSVALSLYPEDAPRNMLPLVCKGEGSLLFEATSLLLWGHTGL
Rat          MTSRDQLVQQVLRNLQEAVESEGLEGLISAALEAKQVLASFSLPICQKGGPGAQVLEVDSVALSLYPEDAPRNMLPLVCKGEGSLLFEAASMLLWGHTGL
Opossum      MISRAQLVQSVLQELQEAAECFGVEGLTTAALEAKKTLSSFSLPSCRSGEQFQGEMDVDSVALSLYPEDAPKNMLPLACKGKGSRLFEAASVLLWGGPGL
Zebra finch  MIQRHQLVQSVLQELQEATECFGLEGLTSAALEAERTLSSFSLPGY-CGSQFQEELEVDRVARSLYPEDAPSNMLPLVCKGEGNRLFEAASVLLWGNPSL
Zebrafish    MIQRAEAVLSVLGELQEATECVGLDALTKVAVEVEQVLAPFPLPTAPCSEIS-SWQGIDAVAHRLYPGDAPTGLLPLVCKGEGNLLFDAASMLLVGSTSL
Tetraodon    MIQRKEVVLSVLGELQEATESSGLNALTRVALEVDQILAPFALPTTPWQDLP-QWACVDEAARDLYPADA------------------------------

                     110       120       130       140       150       160       170       180       190       200
             ....|....|....|....|....|....|....|....|....|....|....|....|....|....|....|....|....|....|....|
Pig          SLELRARTVVEMLLHRHYYLQGMIDSKVMLQAVRYSLCSEESPEMTSLPSATLEAIFDADVKATCFPSSFSNVWHLYALASVLQRNIYSIYPMRNLKIRP
Cow          SLELRARTVVEMLLHRHYYLQGMIDSKVMLQAVRYSLRSEESPEMTSLPSATLEAIFDADVKATCFPSSFSNVWHLYALASVVQRNIYSIYPLRNLKIRP
Horse        SLELRARTVVEMLLHRHYYLQGMIDSKVMLQAVRYSLCSEESPEMTSLPSATLEAIFDADVKATCFPSSFSNVWHLYALASVLQRNIYSIYPMRNLKIRP
Dog          SLELRARTVVEMLLHRHYYLQGMIDSKVMLQAVRYSLCSEESPEMTSLPSATLEAIFDADVKATCFPSSFSNVWHLYALASVLQRNIYSIYPMRNLKIRP
Human        SLELRARTVVEMLLHRHYYLQGMIDSKVMLQAVRYSLCSEESPEMTSLPPATLEAIFDADVKASCFPSSFSNVWHLYALASVLQRNIYSIYPMRNLKIRP
Rhesus       SLELRARTVVEMLLHRHYYLQGMIDSKVMLQAVRYSLCSEESPEMTSLPPATLEAIFDADVKASCFPSSFSNVWHLYALASVLRRNIYSIYPMRNLKIRP
Mouse        SLELRARTVVEMLLHRHYYLQGMIDSKVMLQAVRYSLCSEESPEMTNLSFATLEAIFDADVKATCFPTSFSNVWHLYALASILECNIYSIYPMRNIKIRP
Rat          SLELRARTVVEMLLHRHYYLQGMIDSKVMLQAVRYSLCSEESPEMTNLSFATLEAIFDADVRATCFPTSFSNVWHLYALASILERNIYSIYPMRNVRIRP
Opossum      SSELRARTVVEMLLNKHYYLKGMIDSKVMLQAVRYSLCSEESPEMTSLPLATLEAIFDIDVKATCFPDSFANVWHLYALASVLQHNIYSIYPMWNLKIRP
Zebra finch  SLELQVRTVVEMLLHKQYYLNGMIDSKVMLQAARYSLCTEETPEMTSLPMAILEAIFDADIKATCFPGTFANMWHVYALASVLQCNIYSIYPMSNLKIRP
Zebrafish    SLELQVRAVVEMLIWKRYYLCGMIDSKVMLQAARFSLCAEESQDMLNLPIQVLEAIFDADVKASCFHGSFANMWHVYALASVLQCNIYSVYPMYNLKIRP
Tetraodon    ---LRVRTVVDMVLWKRYYLSGMIDSKMMLQAVRFSLCAEESEDMLNLPVAVLEAIFDADVKASCFPGSYANMWHVYALSSVLQFNIYSIYPMFNLKIRP

                     210       220       230       240       250       260       270       280       290       300
             ....|....|....|....|....|....|....|....|....|....|....|....|....|....|....|....|....|....|....|
Pig          YFNRVIRPRRC--DHMPATLHIMWAG-----QPLTNHLFRHQYFAPVVGLEEVEAESATTSLAPTPPALAPLPPPAKTLELLSQDPGLSYSYLCERYSVT
Cow          YFNRVIRPRRC--DHTPATLHIMWAG-----QPLSGHLFRHQYFAPVVGLEEVEAESAH-------PGPAPLPPPAKTLELLNREPGLSYSHLGEHSSVT
Horse        YFNRVIRPRRC--DHTPATLHIMWAG-----QPLTSHLFRHQYFAPVVGLEEVEAEGTTG-PALAPSALAPLPPPAKTLELLNREPGLSYSHLCERYSVT
Dog          YFNRVIRPRRC--DHVPATLHIMWAG-----QPLTSHLFRHQYFAPVVGLEEVEAEGATG---PAPGPPAPLPASANTLELLNREPGLRYSHLCRRYRVT
Human        YFNRVIRPRRC--DHVPSTLHIMWAG-----QPLTSHFFRHQYFAPVVGLEEVEAEGAPG-VAPALPALAPLSSPAKTLELLNREPGLSYSHLCERYSVT
Rhesus       YFNRVIRPRRC--DHMPSTLHIMWAG-----QPLTSHFFRHQYFAPVVGLEEVEAEGAPG-MAPALPTLAPLSSPAKTLELLNREPGLSYSHLCERYSVT
Mouse        YFNRVIMPRCS--THVTSMLHIMWAG-----QPLTSHLFRHQYFAPVVGLEEVEADCTAS-LNPVPPNLGPLLPPAKTLELLNREPGLSYSHLCDRISIT
Rat          YFNRVIMPRRS--THVTSMLHIMWAG-----QPLTSHLFRHQYFAPVVGLEEVEADGTAS-LDPAPPNLGPLLPPAKTLELLNREPGLSYSHLCDRTSIT
Opossum      YFHRLIRPRCC--NQPPSTLHIMWSGQLLAGQLLACQVFKPEYFSAVVGLEEVEAEVASPPL------PPPPVPPAETLELLNQDPQLTYSHLRERYSIT
Zebra finch  YFNRLIRPRKC--GPRTSTLHIMWSG-----QQLSRQVFKAQYFVAVVGLEELEPTIPSP---------EPPVQPMKTLELLNSDPQLTYSNLRDRYSIT
Zebrafish    YFNRLIRPRIWSKDTEPLTLHIMWSG-----DLEAGSVFKPHKFVALIHASDLKIGSPNS---------EQRMPLVKSLELQNQDTQLSYSNLKNKFNIT
Tetraodon    YFNRVIRPRTRPDGSEPQTIHIMWSG-----VLHSRSLFRPGNFVALVQTGD------DS---------DRQESPGKAEEALSQELQLSYPNLKDKYNIT

                     310       320       330       340       350       360       370       380       390       400
             ....|....|....|....|....|....|....|....|....|....|....|....|....|....|....|....|....|....|....|
Pig          KSTFYRWRRQSQEHRQKVATRFSAKHFLQDSFHRGGVVPLQQFLQRFPEISRSTYYAWKHELLGS-------GTCQALGP------------MEELEKLT
Cow          KSTFYRWRRQSQEHRQKVATRFSAKHFLQDSFHRGGVVPLQQFLQRFPEISRSTYYAWKHELVGS-------GACQALTP------------TEELTKLP
Horse        KSTFYRWRRQSQEHRQKVATRFSAKHFLQDSFHRGGLVPLQQFLQRFPEISRSTYYAWKHELLGS-------GACQALAPKE-------ALAMEELEKLP
Dog          KTTFYRWRRQSQEQRQKVAARFSAKHFLQDSFHRGGVVSLQQFLQRFPEISRSSYYAWKQELLGS-------GTCPALAP-------------------Q
Human        KSTFYRWRRQSQEHRQKVAARFSAKHFLQDSFHRGGVVPLQQFLQRFPEISRSTYYVWKHELLGS-------GTCPALPPRE-------VLGMEELEKLP
Rhesus       KSTFYRWRRQSQEHRQRVAARFSAKHFLQDSFHRGGVVPLQQFLQRFPEISRSTYYAWKHELLGS-------GTCPALPPKE-------VLGMEELEKLP
Mouse        KSTFYRWRRQTQEHRQKVATRFSAKHFLQDSFHRGGFVPLQQFLQRFPEISRSTYYAWKHELLGS-------GANSALGPATPSRE---ALAVPEVERPP
Rat          KSTFYRWRRQTQEHRQKVATRFSAKHFLQDSYHRGGFVPLQQFLQRFPEISRSTYYAWKHELLGS-------GANSALVPATPSRE---AVAVPEVERPP
Opossum      KSTFYRWKRQSREHRQKAATRFVAKHFLQSCFQDGNIIPLQRFRQMFPEISRSTYYAWKHELQGTSD-----GGVSKDPMSG----------ASLEVEEL
Zebra finch  KSTFYRWKRQSREHRQKAAARFAAKHFLQSCFQEGNIIPLQHFRQMFPEISRSTYYAWKHEMQSMVN-----GDTSALSEAHRLQELPRVVPKKADVNEP
Zebrafish    KSTFYRWKRQSIEYHKKSVARYEAKHFFLTSYKQGKLIPLSQFKELFPEIPRSTYYAWKQELVSCCS--LSGGSTGELSPGDSTEQDYWSSPEVKKTPG-
Tetraodon    KRTFYRWKRQTQEHCKKSAARYEAKHFLQACYLEGKLMPLHQFKKFFPEISRSSYYNWKHELLKTGGSFSTSLSTGEVSPGESTEQEAWSSPEGRQEEQE
```

```
                       410       420       430       440       450       460       470       480       490       500
              ....|....|....|....|....|....|....|....|....|....|....|....|....|....|....|....|....|....|....|....|
Pig           EEQVAEG-LGCSSPAVSSPG---MVLMQRAKLYLEHCISLNTLVPYRCFKRRFPGISRSTYYNWRRKALRRNPSFKPAPV-LSVAGATQPASVGEKALLP
Cow           ERQVAEG-LGCSSTAASSPG---VVFMQRAKLYLEHCIALNTLVPYRCFKRRFPGISRSTYYNWRRKALRRNPSFKPVPA-LLESGPPQPVPVGEKALLP
Horse         EEQVVEG-LGCSSLAVSSPG---MVLMQRAKLYLEHCISLNTLVPYRCFKRRFPGISRSTYYNWRRKALRRNPTFKPPPA-LPVAGSPQPTSIGEEALLP
Dog           EEQGAKG-LGGSS-RPSSPG---VILMQRAKLYLEHCISLNTLVPYRCFKRRFPGISRSTYYNWRRKALHRNPSFKPAPA-LSTPGAPQPASVPEEALLP
Human         EEQVAEEELECSALAVSSPG---MVLMQRAKLYLEHCISLNTLVPYRCFKRRFPGISRSTYYNWRRKALRRNPSFKPAPA-LSAAGTPQLASVGEGAVIP
Rhesus        EEQVAEEELECSALAVSSPG---MVLMQRAKLYLEHCISLNTLVPYRCFKRRFPGISRSTYYNWRRKALRRNPSFKPAPA-LSAPGTPQPASVGEGAVIP
Mouse         GKKAAEE-VGCSSLAAAMLSPPSMILMQRAKSFLEYCISLNKLVPYRCFKCRFPGISRSTYYNWRRKALRRTPSFKLSQAAFETAESLQPTDVGKETPFS
Rat           GKMAAEE-LGCSPLTAAISSPTSLILMQRAKSYLEHCISLNKLVPYRCFKCRFPGISRSTYYNWRRKALRRTPSFKLSPG-LETTESLQPTDVGEESLHS
Opossum       EKLSAEGKKSGP-LDPGPMG----VFMQGAKSYLERCISMNTLVPYRCFKRSFPGISRSTYYNWRRKAIKGNPNFKTPPS---FSGGSFHPPPGE-VLHP
Zebra finch   ANPQGSLKSSSPSPDPIQAG----IFMQGAKSYLEKCISMNTLVPYRCFKRSFPGISRSTYYNWRRKAIKENPNFKHPQS---PLDNQKTLAIGK-QFLQ
Zebrafish     -QGSFANMLAFKYEKLEGERNQNVTLMQEAKKSLQDCIVANTSFPYRIFKRKFPGISRSTYYNWRREAMLFIP-FKEHSG------SSEEGSDADKSQSP
Tetraodon     HHDSVAGMFGLSLSKLELERAHSVANMQEAKRCLQSSIATNTSLPFRVFKKNFPGISRSTYYNWRREAMLFTRGYKGGLA------SSEDSSDADKCQSP

                       510       520       530       540       550       560       570       580       590       600
              ....|....|....|....|....|....|....|....|....|....|....|....|....|....|....|....|....|....|....|
Pig           WEG-EVGEEAGKATGGGQPAPREFLPLRMPLSRWQRRLRREARKQVLSGHLPFCRFRLRYPSLSPSTFWVWKSLARGWP---------------------
Cow           WKGGEVGEGAAKATGGGPPAPRAFLPLRVPLSRWQRRLRRAARKQVLGGHLPFCRFRLRYPSLSPSSFWVWKSLARSWP---------------------
Horse         WKG-EVGEGAAKAMGRGPPAPREFLPLRMPLSRWQRRLRRAARKQVLSGHLPFCRFRLRYPSLSPSAFWVWKSLARGWP---------------------
Dog           WKS-KVGEGAGKATAGGPPAPHEFLPPKVPLSRWQRRLRRAARKQVLSGHLPFCRFRLRYPSLSPSTFWAWKSLARSWP---------------------
Human         WKS-EAEEGAGNATGEDPPAPGELLPLRMPLSRWQRRLRRAARRQVLSGHLPFCRFRLRYPSLSPSAFWVWKSLARGWP---------------------
Rhesus        WKS-EAEEGAGKATDEEPPTPQELLPLRMPLSRWQRRLRRAARRQVLSGHLPFCRFRLRYPSLSPSAFWVWKSLARGWP---------------------
Mouse         LKREAGEEETGKAGSGAPLTSRGLISPKMPLSRWQRRLRRAARKQVLNGHLPFCRFRLRYPSLLPSTFWVWKSLSRRSPGMQIPSLSQR-----------
Rat           LKSEVGEEGAGKAVGEAPLTSRGFTSPRMPLSRWQRRIRRAARKQVQSGRLPFCRFRLRYPSLLPSTFWVWKSLSRGRPRMQAPSLSQRKQKLEERQKPE
Opossum       GK----GIPVRKGLAEVQPSARGLLPLKPSLARWQKRLRRAARRQVQSGYLPFCRFRLRYPSLSPTSFWLWKGTAKGRP---------------------
Zebra finch   LKP--SSVPVKKRRNGDRPASRSLLQLHPSLC-WRKQLRDVARRQVQQWQLPFCKYRLRYPSLSSTAYWFWKGSGQITW---------------------
Zebrafish     RSQMSPSKFDRQKLSPRVRISRRKHRSLRLAYHHRKMLREEAKMHIRRSKMSFIKFKLKFPTVSSSFYWLWRVSLNRKP---------------------
Tetraodon     -SSLSP--VPPQPAPPRARVCRSRHRSFRLAYMSKKQLRDAAKLHVQKSKWSLMKFRLRFPTTSPCFYWLWRGSLSRGR---------------------

                       610       620       630       640       650       660       670       680       690       700
              ....|....|....|....|....|....|....|....|....|....|....|....|....|....|....|....|....|....|....|
Pig           --------------RSLSKLHIQAPTLGKGGIQEVEEKQEKEAGRNVTAAMAPPAGT-LQMTASPGEDPGAALGGPSREG-ALQEGAMAQGRP-------
Cow           --------------GSLSKLHIPAPTLGRGSRKEAEEK---EAGRNVIAAVAPPEGT-WPMAASPGQDPRKATGGPSREG-ALQEGPTAQGRPPSGSLSS
Horse         --------------RSLSKLQMQASTLGKAGVQEAGEKREKEAGRNMAAAMTPPAGT-PKVGASPGEDPGKAEGGPSREG-VLPGAATAQGRPHSGSLSS
Dog           --------------RNLSKFQMQAPTLGKVGMQAAEEKQEKEAGGDVIAVNVPPAGTPLQVGASPGEDPGKAQEGPSREG-AIT-----QGQPHSRFLSS
Human         --------------RGLSKLQVPVPTLGKGG-QEAEEKQEKEAGRDVTAVMAPPVGA-------SSED---VEGGPSREG-ALQEGATAQGQPHSGPLLS
Rhesus        --------------RGLSKLQVPAPTLGKGG-QEAEEKQEEEAGRDVTTVMAPPVRA-------SSED---AEGGPSREG-ALQEGATAQGQPHSGPLLS
Mouse         -RQKPQGRQKPEGRQKPEEQQKPEGRQKPEGRQKPVEPQAMEADQNVPAMVVPPAET-LPVATSPED----VPGGPSREGNIIQEAAMTQSQPHSGSLPS
Rat           ERQKPEEWQKPEERQKPEEWQKPEERQKPEERQKPVEWQDVEAVRKVTPVVVPSVET-LPVAASPEDAPEIAPGGPSREG-IVQEAAVVQGQPHSGSLPS
Opossum       ----------------NSRFNHP-SQIPNKEILGAAG-KAEVGGKPVLPSVEKEAAP-------------------REDRSLGPYHGTLSGYAVAGGGGS
Zebra finch   ----------------QHRLPWSSSSVPLNRVASLAPPRAEPGLKSQCHLEVATKHI-------------------DKPVPAVPCNTTISGYAMS-----
Zebrafish     --------------------------EKPAVMSNSEESKSLAISPDVFQKIKTQGMF-------------------SFDG--NIDCLNGQTEFTMP-EYS
Tetraodon     --------------------------KVAEEDRLPAGGRDPAAESTSVARVPTSHAP-------------------RGKDPQPLQRPSAPSSDAPPSKHA

                       710       720       730       740       750       760       770
              ....|....|....|....|....|....|....|....|....|....|....|....|....|....|....|....
Pig           ----MVAAAGGRDGQVLVMDMLATTKFKAQAKLFLQKRFQSKSFPSYKEFSALFPLTARSTYYMWKRALYDGLTLVDG*
Cow           HPVVTAAAAGGRDGQVLVMDMLATTKFKAQAKLFLQKRFQSKSFPSYKEFSTLFPLTARSTYYMWKRALYDGLTLVDG*
Horse         RP--VVAVAGGRDGQVLVMDMLTTTKFKAQAKLFLQKRFQSKSFPSYKEFSALFPFTARSTYYMWKRALYDGLTLVDG*
Dog           HP-VAEAAAGGRDGQVLVMDMLATTKFKAQAKLFLQKRFQSKSFPSFKEFSALFPLTARSTYYMWKRALYDGLTLVDG*
Human         QP--VVAAAGGRDGRMLVMDMIATTKFKAQAKLFLQKRFQSKSFPSYKEFSALFPLTARSTYYMWKRALYDGLTLVDG*
Rhesus        QP--VVAAAGGRDGRMLVMDMIATTKFKAQAKLFLQKRFQSKSFPSYKEFSALFPLTARSTYYMWKRALYDGLTLVDG*
Mouse         QT--LAEAPGGSDGQVLVMDMLTTTRFKAQAKLFLQKRFQSKTFPSYKEFQALFPLTARSTYYMWKRALYEGLTLIDG*
Rat           QT--MVMAPGGSDGQVLVMDVLSATRFKAQAKLFLQKRFQSKTFPSYKEFQALFPLTARSTYYMWKRALYEGLTLVDG*
Opossum       GG-----GDSNNNGQVFVMDVIATTQFKAQAKLFLQRRFESKTFPSYKEFSAHFPFTARSTYYMWKRALHDGLTLVDA*
Zebra finch   ---------ERANSRMFVMDVIATAQFKAQAKLFLQQRFESKTFPTYKEFSARFPLTARSTYYMWKRALHDGLTLVDA*
Zebrafish     PP------DQSTNDHMFVMDVVALANFKAKAKLFLQQRFEEKAFPTFKEFRSYFPLTPRSTYYMWKRALHHGVPLIHA*
Tetraodon     HP------KVSLNQQMFVMDVVALANFKAKAKLFLQQRFEQKAFPTFKEFRSYFPFTPRSTYYMWKRALYHGVSLIHG*
```

B

Pig	*Sus scrofa*		
Cow	*Bos taurus*	XP_607139	89.0%
Horse	*Equus caballus*	XP_001490454	90.1%
Dog	*Canis lupus familiaris*	XP_547904	86.8%
Human	*Homo sapiens*	NP_060698	88.6%
Rhesus	*Macaca mulatta*	XP_001093846	88.4%
Mouse	*Mus musculus*	NP_001028948	74.5%
Rat	*Rattus norvegicus*	XP_001059118	74.5%
Opossum	*Monodelphis domestica*	XP_001375329	63.7%
Zebra finch	*Taeniopygia guttata*	XP_002199939	56.9%
Zebrafish	*Danio rerio*	NP_001070608	45.7%
Tetraodon	*Tetraodon nigroviridis*	CAG01823	45.3%

C

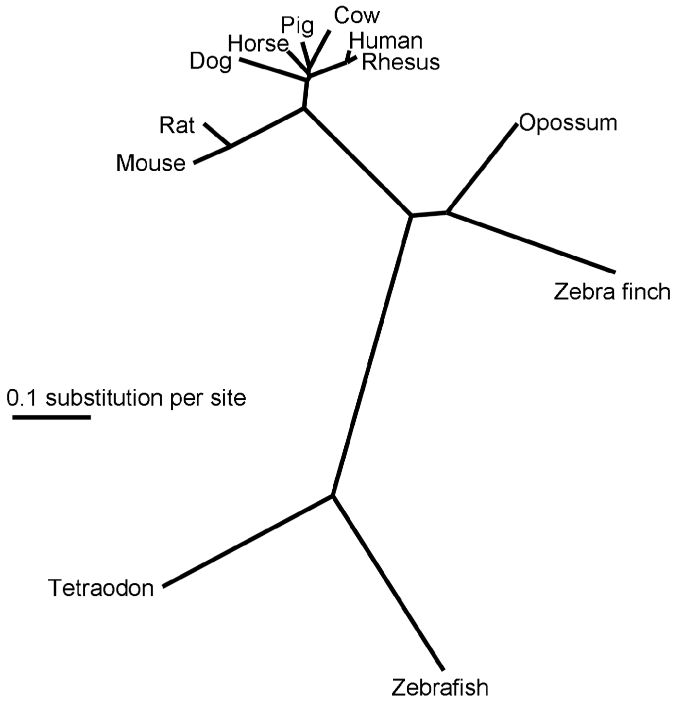

Supplement: Additional file 9 — Figure S5: Orthologs of swine vertnin protein. A. In the public database, protein sequences probably coded by orthologous genes of swine VRTN were found not only in mammals, including opossum, but also in a bird (zebra finch) and fish (zebrafish and tetraodon). The orthologous genes were conserved at the start and stop codons, so it seems that VRTN encodes a functional protein. Alignment was performed with ClustalW software. Underlines in the swine sequence indicate the homologous region to helix-turn-helix domain of Transposase IS3/IS911. B. The identities (%) of amino acid sequences to swine VRTN. C. Phylogenic tree of vertnin proteins constructed with ClustalW software. [file 1471-2156-12-5-S9.PDF]
